# Gleanings from Our Exchanges

**Published:** 1874-01-01

**Authors:** 


Foreign Bodies in the Stomach.—A case is recorded in *Il Raccoglitore Medico* (No. xvi., 1873), by Dr. Benedetti, in which a nun, aged twenty-two, after suffering for some days from symptoms of gastric fever, with obstinate vomiting, ejected from her stomach a brass cross, one-third of an inch long, the cross-piece being one-fourth of an inch long. She remembered having swallowed it when she was nine years old. In the interval it had not produced any inconvenience. A case is also related in the *Imparziala* for June, in which a soldier swallowed a tablespoon. Severe dyspnœa followed; and in about three-quarters of an hour the spoon was ejected by vomiting.—*London Medical Record.*

ELECTRO-THERAPEUTICS. — The morbid conditions wherein electro-therapeutical applications are indicated may be briefly summed up, viz.: in their efficacy in restoring normal action in partial or general paralysis, or wherever there is great atrophy or inert muscular action, dependent upon deficient nervous tone or deranged action in the nervous centers; in the subjugation of the violent pain of articular rheumatism; in certain atonic or debilitated conditions of the system, owing to impaired nutrition; in the removal of malignant tumors where surgical interference is entirely out of the question, and especially in the extirpation of soft, morbid growths, etc.; and the effects of electrolysis and galvanism have been successfully demonstrated upon anatomical secretions, excretions and morbid cell developments. In applying electricity to tumors we use electrodes with sponge tips, saturated with a strong solution of chloride of sodium. We always commence from the border of the tumor and gradually approach the more solid particles; as a result, suppuration and absorption set in and destroy the growth.

In this connection we beg leave to refer to a case which came under our own treatment:

Dr. G., a prominent Catholic clergyman, of Brooklyn, N. Y., who suffered from malignant tumor, involving the right side of the face, neck, trachea, and other vital organs, to such an extent as to be unamenable to surgical treatment. He had consulted prominent members of the profession, without encouragement or success. The growth was rapid and pointed to an early dissolution by suffocation, presenting all the characteristics of schirrus. In June, 1871, he called at our office to consult us concerning his trouble, whereupon we informed him of its serious and threatening nature, and that, in our opinion, little encouragement could be given, save that which might be afforded by the judicious use of electricity. Our mode of treatment in this case was daily application of the galvanic current, from a sixteen-cell battery (of G.-F. Manf. Co.) This treatment was continued from June 5th to July 7th, twenty-four applications in all, with great success, the tumor having entirely disappeared, relieving him of all its distressing features.—*Dr. Turnbull, Phil. Med. and Surg. Reporter.*

---

ENORMOUS ACCUMULATION OF EXTRANEOUS MATTER IN THE STOMACH.—The patient in this case was a girl, aged four, who died in Hardwicke Hospital, of uncontrollable purging and vomiting. For nine months she had had a hard tumor in her abdomen, but nothing peculiar had been observed by her mother about her appetite. She never complained of any pain till two days before her death, when she was attacked with sudden colic, which lasted about an hour and recurred twice. The tumor was large and hard, and was freely movable. At the autopsy it was found to be composed of a collection of extraneous matters, such as pieces of cloth, cord, straw, grass, chips of wood, etc., which were all matted together into one large mass occupying the entire cavity of the stomach. A similar aggregation was found near the end of the jejunum. The rest of the intestine was empty and healthy throughout, with the exception of a large ulcer which existed in the duodenum. Dr. Yeo, by whom the specimen was shown at the meeting of the Dublin Pathological Society, considered the case interesting as showing the difficulties attending the diagnosis of the disease called *pica*, when the peculiar aberation had never been observed. The amount and position of the aggregation was also unusual. The ulcer in the duodenum seemed to support the idea that some irritation in the alimentary tract is the usual cause of the disease.—*Medical Press and Circular. Richmond and Louisville Journal.*

---

INTENTIONAL FRACTURE OF THE FEMUR TO EQUALIZE THE LENGTH OF THE LEGS.—Professor Rizzoli, of Bologna, has treated with entire success four cases of shortened femur, by fracturing the bone of the sound limb and shortening it to the same length as the other. In one case, a girl, prior to the operation, scarcely touched the floor with the great toe of the shortened limb. The *N. Y. Med. Record*, on mentioning the case, refers to a surgeon of New York, though without naming him, who not long since exsected a portion of the femur in a sound thigh, for the purpose of shortening it to an equal length with the other femur. This was prior to the European operation.

MODIFICATION OF THE OPERATION FOR HARE-LIP.—On two successive Saturdays Sir William Fergusson has recently demonstrated to the students of King's College, London, a novel modification of the ordinary operation for hare-lip. The cases in which he carried out this plan were of the usual type, the fissure being, as in the majority of instances, on the left side; and in both it was considered advisable to take away the intermaxillary bone. This adds to the success of the operation, not only by removing an occasional obstacle to primary union, but because the teeth, which are subsequently developed from the protecting knob, are worse than useless, by reason of their deficient development and faulty position. Instead, however, of removing the portion of bone readily with the knife or bone-forceps, it had occurred to Sir William that it would be much better to operate subcutaneously, so to speak, by stripping off and retaining the mucous membrane; and accordingly this was done, with perfect success, the bone shelling out readily from its investment. Not only will this procedure greatly accelerate the subsequent process of healing, but the advantage is obvious, of retaining a thick and firm mucous surface in preference to the more artificial substitute of cicatricial tissue.—*Brit. Med. Jour.*, Nov. 1, 1873.

---

NEW MEANS OF DILATION IN STRICTURE OF THE URETHRA.—It simply consists in the employment of a column of liquid about twenty metres high, established by means of a funnel, and containing about a pound and a half of water (boiled at 25 deg. or 27 deg. C.), and suspended above the patient's bed. An India-rubber tube (about two metres long), and provided with a cock in the middle of its length (so as to moderate or suspend the current of water), and having at its end a small glass pipe like an ordinary syringe, which is to be introduced into the meatus urinarius, connects the apparatus with the penis. The glass end being introduced, the cock is more or less opened at will, and slight pressure is exerted on the glands, to prevent the water from running outside. The water in the funnel is then forced down by its own weight, and runs down drop by drop, dilating the stricture without pain, and through its local antiphlogistic action, rendering the urethra pervious to sounds and bougies. The patient can himself apply the apparatus three or four times a day, and when it is removed, the surgeon has only to make use of his sounds or bougies.—*Mouvement Medical.*

---

DRY CUPPING INSTEAD OF TAXIS IN HERNIA.—I have abandoned the taxis in hernia; a large cup is applied to the abdominal wall; an assistant is directed to stand in between the legs of the patient, and elevate the hips as high as possible, while I produce a large vacuum, by traction on the abdominal wall with my cup, and the weight of the bowel retained, together with the atmospheric pressure, cause the protruded portion to slip backward to its appropriate place very readily. I have not used the taxis for twenty-five years, as this plan is, by far, preferable.

As an alterative in amenorrhœa, dysmenorrhœa, and leucorrhœa, it cannot be surpassed. While practicing in Logan County, Kentucky, I cured a case of leucorrhœa by dry-cupping and cool water injections, in the space of three months, which had defied the prescriptions of Dr. Boas Roberts and other physicians for eleven years.—*Dr. B. H. Washington in Nashville Med. Journal.*

---

MEANS OF DIAGNOSTICATING LIPOMATA.—A character peculiar to lipomata resides in the property belonging to all fatty tumors of hardening under the action of cold. When, after the use of ice or the ether spray, in the case of a doubtful tumor, the growth is felt to become harder, the presumption is that the case is one of lipomata.—*Rev. Med. Phot. des Hopiteaux.*—*Richmond ana Louis. Jour.*

TREATMENT OF DIPHTHERIA.—Dr. Lolli strongly recommends the following measures, having found them extremely efficacious in a great variety of cases: First, avoid cauterizing, except when gangrene occurs. Second, do not have recourse to bleeding, purging, or emetics, unless you are forced to do so by exceptional symptoms. Third, diet according to appetite; but at all events generous. Fourth, do not interfere with the functions of the skin; or rather promote them by rest in bed, poultices, sinapisms, etc., and persevere until, from the general and local symptoms, it may be supposed that the morbid principle has been eliminated or destroyed. Fifth, use the following mixture as a gargle, or apply with a camel's-hair pencil every second hour, or employ as an inhalation, if the disease has reached the larynx:

℞—Lime-water, f. ℥ iv. to xij.

Sol. of perchloride of iron, f. ʒ ss. to f. ʒ ij.

Carbolic acid, gr. j. to xx.

Honey of rose, f. ℥ j.

Shake bottle well. The mixure may be largely diluted with water or tea, and given internally.—*Lancet.*—*Peninsular Journal.*

---

TREATMENT OF ENURESIS. By Dr. Buyelmann, Cologne.—The author was induced by an article in the Berlin *Klin. Wochenshrift*, 1871, No. vi., to try the syrup ferri iodidi in a severe case of incontinentia urinæ. The patient was a young girl, thirteen years old, of nervous temperament, and anæmic. The principal complaint was the incontinence of urine, so severe as to prevent her from walking any distance from her home without wetting herself. In addition to generous diet, she took, for three weeks, syrup ferri iodidi, seven grammes ad aquæ, syrup simp., aa, fifty grammes; a teaspoonful every two hours. After a week's treatment, there was a marked improvement, and, in two weeks more, she was discharged well.—*Berlin Klin. Wochenshrift*, No. vi.—*Richmond and Louisville Journal.*

CHLORIDE OF POTASSIUM IN EPILEPSY.—Dr. Lander uses chloride of potassium instead of bromide of potassium in epilepsy. He mentions the following advantages in the employment of the substance: "It is more active, is but one-sixth of the cost, and has not the secondary effects of the bromide. He begins with small doses, but has been able to continue the use of the substance for months without any inconvenience, in daily doses of from one drachm to a drachm and a half. According to Dr. Lander, bromide of potassium is transformed into the chloride in the stomach. This is therefore an additional reason for prescribing it at once in this latter form."—*Scalpel, Belgium.*—*Peninsular Journal.*

---

"THE LANCET" JUBILEE.—With the number for October 4, 1873, *The Lancet* completed the fiftieth year of its existence. The energy and talent of its founder, the late Mr. Wakley, enabled it to weather the severe storms which darkened its earlier career, and have given to it a position of influence and usefulness which is rarely accorded to any periodical. Age has served to develop the strength of *The Lancet*, and render it even more worthy of the patronage and support which, as one of the leading medical journals of Great Britain, it has for many years enjoyed.

---

SUGAR AND MAGNESIA AN ANTIDOTE TO ARSENIC.—The *Mouvement Medical* relates various experiments conducted by Mr. Carl, with the result of showing that sugar, mixed with magnesia, may serve as an antidote in cases of poisoning by arsenious acid, in which cases, too, the internal use of the hydrated magnesia is most valuable.—*Lancet.*

---

AN interesting case is reported by M. Dieulafoy in which an infant, six hours old, was poisoned by a dessertspoonful of laudanum, and from whose stomach the poison was extracted, before it had taken fatal effect, by means of the pneumatic aspirator.—*Ex.*

JOHN AUBREY, who was at Harvey's funeral and "helpt to carry him into the vault," writes: "I have heard him (Harvey) say, that after his 'books of the Circulation of the Blood' came out, he fell mightily in his practice, and 't was believed by the vulgar that he was crack-brained; and all the physicians were against his opinion and annoyed him. All his profession would allow him to be an excellent anatomist, but I never heard of any that admired his therapeutique way. I knew several practitioners in this town (London) that would not have given 3*d* for one of his bills (prescriptions); and that a man could hardly tell by one of his bills what he did aime at."—*Ex.*

---

DR. JOSEPH BELL, of Edinburgh, in a paper on surgical cases in relation to temperature, lays down the following axioms:

1. Suppuration, even very profuse, does not necessarily imply any great rise in temperature, so long as it is not putrid. 2. Fœtor or putrefaction of suppuration always induces a rise in temperature. 3. A high temperature, lasting for more than three or four days after the injury or operation, indicates mischief impending, such as sloughing or abscess. 4. The temperature generally gives warning a day, or even two days, before the pulse.—*Richmond and Louisville Jour.*

